# Enriching the evidence base: moving towards full societal costs and benefits

**DOI:** 10.1186/1472-6963-14-S2-O38

**Published:** 2014-07-07

**Authors:** Werner Brouwer

**Affiliations:** 1 iBMG, Erasmus University Rotterdam, The Netherlands

## 

Economic evaluations are a form of applied welfare economics and aim to inform policy makers about the impacts on social welfare of particular policies. It is clear that in light of this aim, economic evaluations need to include all relevant societal costs and benefits related to a policy. Only then, a full welfare economic trade-off between costs and benefits can be made to facilitate welfare improving decision making.

It is clear that current economic evaluations in the field of health care commonly do not include all relevant costs and benefits related to interventions. In many jurisdictions, a health care perspective is prescribed, which precludes the inclusion of relevant societal impacts, such as productivity costs, costs of informal care and costs in other sectors.

Also in countries prescribing or allowing broader perspectives, the inclusion of broader costs and benefits related to health care interventions are often ignored. This partly is related to a lack of methodological development and consensus.

In this presentation, I will highlight some of the main, often ignored, categories of societal costs and benefits, also touching on the methodological development in measuring and valuing these. I will also briefly address the need for investigating the monetary value of health and for inclusion of equity considerations in economic evaluations.

Only by enriching the evidence base provided by health economic evaluations can these ultimately contribute to welfare improving decisions.

